# IMPC impact on preclinical mouse models

**DOI:** 10.1007/s00335-025-10104-4

**Published:** 2025-01-16

**Authors:** Sabine M. Hölter, Pilar Cacheiro, Damian Smedley, K. C. Kent Lloyd

**Affiliations:** 1 Institute of Experimental Genetics and German Mouse Clinic, Helmholtz Munich, German Research Center for Environmental Health, Neuherberg, Germany; 2 Technical University Munich, Munich, Germany; 3 German Center for Mental Health (DZPG), Partner Site Munich, Munich, Germany; 4 Faculty of Medicine and Dentistry, William Harvey Research Institute, Queen Mary University of London, Charterhouse Square, London, EC1M 6BQ UK; 5 Department of Surgery, School of Medicine, University of California Davis, Sacramento, CA USA; 6 Mouse Biology Program, University of California Davis, Sacramento, CA USA

## The challenge

Complete sequencing of genomes for human, mouse, and several other species was a technological breakthrough that identified and mapped thousands of genes and non-coding regions, much of which had heretofore been unknown. But it soon became apparent that significant knowledge gaps existed in understanding the in vivo function of most of these genes. Scientific research progress to address this deficiency was painstakingly slow and arduous, resulting in only partial functional annotation of a small number of well-characterized genes and gene sets. This self-fulfilling research paradigm overlooked genes with little to no known function, leaving in its wake a neglected “dark” genome. To accelerate progress and reveal gene function and insights into genetic associations and causes of disease, a fundamental shift from incremental steps to transformative change was needed. In response, a collaborative, global initiative emerged to systematically generate and phenotype a comprehensive collection of genetically modified “knockout” mouse models. This mandate was adopted and implemented by the International Mouse Phenotyping Consortium (IMPC) (Brown and Moore 2012a, b), a network of 21 academic research institutions across 15 countries on 5 continents, including leading laboratories from Europe, North America, Asia, and Africa. IMPC members agreed a mission to “create a comprehensive catalog of mammalian gene function that is freely available for researchers” by producing mouse models with targeted disruptions of every human orthologous protein-coding gene in the mouse genome. These knockout models have been and continue to be subjected to a standardized series of phenotyping assays across multiple body systems (Brown and Moore 2012a, b; Brown et al. 2005), allowing for identification of key biological processes and functional pleiotropy (Brown and Lad 2019), sexual dimorphism (Karp et al. 2017; Wilson et al. 2022), and essentiality for each gene (Cacheiro et al. 2020). Depositing mice and data into publicly accessible repositories are making these resources available for researchers around the world to extend this new knowledge into studies of the genetic effects on specific disease mechanisms. These efforts aim to accelerate disease diagnoses, identify new druggable targets, develop novel therapeutic interventions, and enact effective disease prevention strategies (Groza et al. 2023).

## The impact

**Fig. 1:**
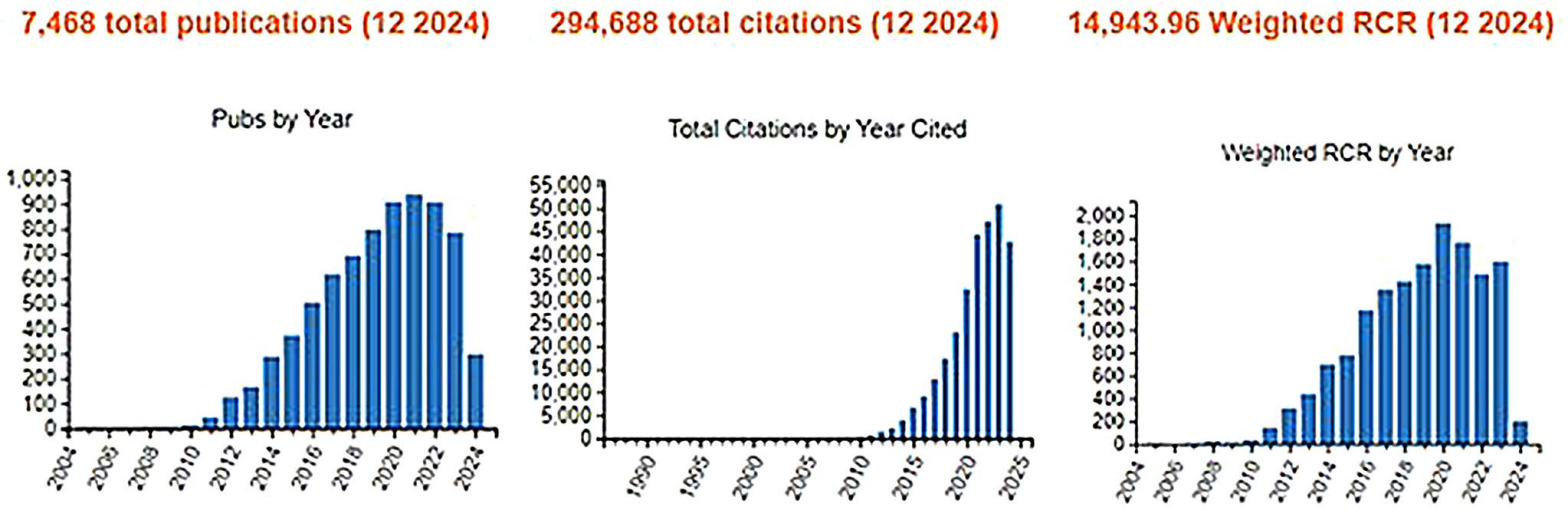
Influence of IMPC publications according to NIH iCite (https://icite.od.nih.gov) on 5 December 2024

To date, data emerging from the study of IMPC mice has become an invaluable scientific resource for the biomedical research community, facilitating the study of gene function and the identification of novel therapeutic targets for human diseases. The vast phenotypic data generated not only in the project consortium itself but also by the greater biomedical research community using IMPC-generated mouse models and data has substantially enhanced our understanding of gene-disease relationships and genetic influences on mechanisms of disease. A publication tracking system using natural language processing methods, followed by annotator reviews through an IMPC-specific literature monitoring and curation tool (Cacheiro et al. 2024), identified nearly 7,500 papers that have used IMPC mice, data, and/or biomaterials to expand preclinical knowledge on a variety of diseases and disorders, including cardiac dysfunction (Guo et al. 2018; Wang et al. 2019; Spielmann et al. 2022), schizophrenia (Mihali et al. 2012; Lago and Bahn 2022; Garrett et al. 2024), Alzheimer’s (Rao et al. 2020; Cheng et al. 2021; Wang et al. 2021), ciliopathies (Wang et al. 2020; Higgins et al. 2022), osteoporosis (Swan et al. 2020; Formosa et al. 2021; Stein et al. 2023), metabolic syndrome (Ng and Gloyn 2013; Rozman et al. 2018; Andersen et al. 2022), hearing loss (Bowl et al. 2017; Trpchevska et al. 2022), developmental conditions(Dickinson et al. 2016; Dhombres et al. 2022), ophthalmic disorders (Khaled et al. 2019; Chee et al. 2023; Fritsche et al. 2016), dermatopathologies (Morell et al. 2022), and others. As shown in Fig. 1, according to NIH iCite, a digital tracking tool for citations (Hutchins et al. 2019a, b), influence (Hutchins et al. 2016, 2017) and the prediction of translational progress (Hutchins et al. 2019a, b), the exact number of 7468 IMPC-related publications from 2005 to 2024 have resulted in a total of 294,688 citations. These publications originated either from consortia contributing to the development of IMPC like EUMORPHIA (Brown et al. 2005), EUCOMM (Friedel et al. 2007), EUMODIC and Sanger MGP (Hrabe et al. 2015; Ayadi et al. 2012), from the IMPC itself, or from researchers using IMPC resources. Altogether they resulted in a weighted relative citation ratio (RCR) of 14943.96, more than double the number of total publications, indicating a highly influential set of articles. These achievements, and surely more to come, will fuel successful research grant applications and publication of more scientific papers on an even greater variety of disease topics in the future, especially as newer data are added to the growing catalogue of mouse models for more genes in the remaining years of the project.

An expanding community of users and growing numbers of papers indicate that the IMPC is fulfilling expectations and delivering new scientific knowledge about genes and gene function that is useful for the scientific community. While this demonstrates the IMPC has been effectively illuminating the previously dark genome, these metrics do not reflect the translational impact of IMPC. For example, how has IMPC inspired clinical insights, catalyzed the accuracy and speed of disease diagnoses, accelerated the identification of novel druggable targets, led to the development of new or repurposing of existing therapeutics, or validated effective preventative strategies. These are very difficult measures to assess. An analysis using the translation module of NIH iCite that predicts the translation of scientific knowledge into clinical studies (Hutchins et al. 2019a, b) reveals that 789 of the 7468 IMPC publications mentioned above have been cited in clinical publications. Indeed, by identifying and characterizing disease-associated genes, IMPC resources are contributing to the development of preclinical models with predictive value for eliminating diagnostic odysseys, enhancing drug discovery, and reducing disease incidence. As a platform for translating newly revealed molecular mechanisms underlying diseases to impacts on human health, IMPC resources have contributed significantly to improvements in the diagnosis, treatment, and prevention of a wide range of conditions, from rare genetic disorders to common diseases like cancer, cardiovascular diseases, cognitive decline, and metabolic disorders.

Moreover, phenotype observations from IMPC knockout mice have directly impacted clinical medical practice, especially in personalized medicine. Our recent analysis highlighting the value of the IMPC for human genetic studies found that the resource has been implicated in at least 109 validated rare disease–gene associations over the last decade (Cacheiro et al. 2024). In addition, knockout models of human disease-associated genes have been crucial for evaluating the efficacy and safety of new therapies and improving the precision of treatments tailored to individual genetic profiles of patients. The public health impact of IMPC is also substantial in that it fosters the development of new diagnostic tools and therapies that can address unmet clinical needs of patient populations. Specific examples for this usage of the IMPC resource in preclinical research are described in the following paragraphs.

## Specific examples

The IMPC has facilitated the development of new tools for diagnosing, managing, and preventing complex diseases, as studies using IMPC mice have led to discovery and characterization of potential biomarkers that could be used for early detection and diagnosis, monitoring disease progression, prognostication, and the development of personalized therapeutic strategies. For example, studies using *Prkcb* knockout mice support PKC-beta as a potential new drug target for the treatment of high-fat induced non-alcoholic fatty liver disease (Shu et al. 2021). In addition, mice with low *Tgfbr2* expression are predisposed to spontaneous gastrointestinal tract tumors, suggesting that TGFBR2 could be a potential biomarker for early detection of colorectal cancer in humans (Gough et al. 2021). Experiments using *Bcl2l11* knockout mice showing premature neuronal apoptosis have led to investigations into using BCL2L11 as a biomarker to assess neurodegenerative disorders and cognitive decline in Alzheimer’s and Parkinson’s diseases (Sionov et al. 2015). Further, *Fto* knockout mice showing metabolic dysfunction have prompted studies of *FTO* gene variants as biomarkers for obesity risk in humans (Najd-Hassan-Bonab et al. 2022). Also, altered immune responses and increased susceptibility to lupus-like phenotypes in *Ifnar1* knockout mice have highlighted interferon (IFN) signaling pathways and IFN-induced biomarkers as potential diagnostic markers of autoimmune disease (Ban et al. 2021).

As well as being used for investigating novel biomarkers, IMPC knockout mouse models have also uncovered fundamental biological mechanisms that have accelerated drug discovery pipelines by helping researchers identify new drug targets, validate existing ones, and assess drug efficacy and safety. Experiments using IMPC mice have supported research studies targeting *Tgfbr2* for cancer drug discovery (Gough et al. 2021), *S1pr1* for neurological diseases (Kandjani et al. 2023), *Fto* for obesity and metabolic diseases (Azzam et al. 2022), *Sirt6* for aging and cancer (Akter et al. 2021), and *Prokr2* for obesity and circadian rhythm disorders (Sarfati et al. 2010; Martinez-Mayer and Perez-Millan 2023). Studies on these models have provided preclinical validation of drug targets and help optimize therapeutic strategies.

The IMPC has also provided critical insights into the genetic basis of diseases, identifying key molecular pathways and potential therapeutic targets for precision medicine and pharmacogenomics. Research using IMPC mouse models have demonstrated how genetic alterations influence disease phenotypes, guiding the design of safe and effective treatments that are tailored to individual genetic profiles. For example, drug efficacy in gene-targeted therapeutics have been predicted in studies on *Tgfbr2* knockout mice (Zhao et al. 2024), drug safety associated with genetic variability has been assessed in *Cpd2d6* mice (Taylor et al. 2020), and genetic factors that make individuals susceptible to type 2 diabetes, obesity, and cardiovascular diseases have been identified using *Pparg* mice (Lefterova et al. 2014). With respect to brain research, *Slc20a2* mice have provided a solid preclinical model to study the development and treatment of a rare neurodegenerative disorder of brain calcification (Jensen et al. 2018), *P2rx7* mice can be used to study immunological features and subtypes of depression (Urbina-Trevino et al. 2022), and several IMPC lines are suitable models for drug repurposing studies in schizophrenia (Lago and Bahn 2022). These models have helped researchers better understand the genetic underpinnings of diseases, predicted drug responses, optimized therapeutic strategies, and identified at-risk populations who may benefit from personalized treatments.

For example, *Kmo* (kynurenine 3-monooxygenase), an enzyme in the kynurenine pathway, plays a role in the excessive inflammatory response to pancreatitis which involves release of pro-inflammatory cytokines, oxidative stress, and activation of immune cells (Mole et al. 2016). *Kmo* knockout mice produced by the KOMP2 project reduced levels of inflammatory cytokines like quinolinic acid and prevented the excessive activation of macrophages and neutrophils in an experimental model of acute pancreatitis. Not only did this study provide compelling evidence that the *Kmo* knockout mouse was a valuable tool for understanding genetic regulation of acute pancreatitis, but it also demonstrated that *Kmo* inhibition could modulate metabolic pathways related to inflammation. This finding may be especially relevant in the context of critical illness and organ failure. In this way, these studies have laid the groundwork for exploring *Kmo* as a druggable target for the development of KMO inhibitors to control inflammation, prevent organ damage, and improve survival in acute pancreatitis and other diseases.

Notably, in recent years IMPC has also facilitated research exploring the feasibility and efficacy of gene therapy approaches in a broad range of disease areas. Examples range from genetic forms of deafness or hearing loss and associated vestibular deficits (Michalski and Petit 2022; Ding et al. 2021; Maudoux et al. 2022), restoration of visual function for blue cone monochromacy and retinal degeneration (Deng et al. 2018, 2019; Beryozkin et al. 2021; Qian et al. 2022; Hsu et al. 2023; Lu et al. 2023; Abu-Diab et al. 2023), managing hereditary spastic paraplegia (Hauser et al. 2019; Chen et al. 2023; Lim et al. 2024) and other upper motor neuron diseases like amyotrophic lateral sclerosis (Genc et al. 2022), and treating mitochondrial myopathy (Pereira et al. 2020), muscular dystrophies (Li et al. 2023; Hindi et al. 2023), and cardiac dysfunction (Li et al. 2020; Magadum et al. 2021; Martin et al. 2021; Wingert et al. 2024). Other examples include evaluation of new therapies for lysosomal and glycogen storage diseases (Koeberl et al. 2024; Gardin et al. 2024; Chen et al. 2022; Lim et al. 2020; Vidal et al. 2018; Goodman et al. 2021), neurodegenerative disorders (Jaillard et al. 2021; Lee et al. 2023; Jonquieres et al. 2018), metabolic dysfunctions (Khoja et al. 2018; Pontoizeau et al. 2022, 2024; Sonaimuthu et al. 2021) and mucopolysaccharidosis (Roca et al. 2017). These preclinical studies prompted by IMPC-generated data and resources have inspired the development of treatments for rare genetic diseases for which no treatments currently exist and which are often fatal, with the possibility to significantly impact patients’ lives. The examples above also show that IMPC provides insights not only into rare monogenetic diseases where only limited patient cohorts exist, but also for more common and age-related conditions like blindness, hearing loss, and heart failure. In the latter cases a mouse model can be a useful tool for the development of a therapeutic intervention in preclinical research if it features an aspect of the pathological mechanism that is targeted.

In summary, the IMPC is a cornerstone of translating functional genomics into clinical impact despite its limitations. An obvious limitation is the choice of a single inbred strain to produce all IMPC knockout mouse lines for feasibility reasons. It is well known that phenotypes can change depending on genetic modifiers in different genetic backgrounds, as e.g. already shown for social behaviour (Arbogast et al. 2016), vision (Hoelter et al. 2008) and hearing (Newton et al. 2023), which also implies that the IMPC programme should be considered a sensitized screen. Nevertheless, the mice, data, and biomaterials it has and continues to produce are shaping the future of biomedical research, drug development, and medical practice. These contributions are crucial for making real impacts to advance precision medicine, improve patient outcomes, and enhance public health worldwide.

## Data Availability

No datasets were generated or analysed during the current study.
